# Low-Cost Internet-of-Things Water-Quality Monitoring System for Rural Areas

**DOI:** 10.3390/s23083919

**Published:** 2023-04-12

**Authors:** Razvan Bogdan, Camelia Paliuc, Mihaela Crisan-Vida, Sergiu Nimara, Darius Barmayoun

**Affiliations:** 1 Faculty of Automation and Computers, Politehnica University of Timișoara, 300006 Timisoara, Romania; 2 Research Center for Engineering and Management, Politehnica University of Timișoara, 300006 Timisoara, Romania

**Keywords:** water pollution, water management, low-cost prototype solution, rural area, internet-of-things, sensors

## Abstract

Water is a vital source for life and natural environments. This is the reason why water sources should be constantly monitored in order to detect any pollutants that might jeopardize the quality of water. This paper presents a low-cost internet-of-things system that is capable of measuring and reporting the quality of different water sources. It comprises the following components: Arduino UNO board, Bluetooth module BT04, temperature sensor DS18B20, pH sensor—SEN0161, TDS sensor—SEN0244, turbidity sensor—SKU SEN0189. The system will be controlled and managed from a mobile application, which will monitor the actual status of water sources. We propose to monitor and evaluate the quality of water from five different water sources in a rural settlement. The results show that most of the water sources we have monitored are proper for consumption, with a single exception where the TDS values are not within proper limits, as they outperform the maximum accepted value of 500 ppm.

## 1. Introduction

Water is an indispensable source for life, economic development, and balance in the natural environment. These are some reasons why the preservation of its quality is of utmost importance for humankind. There are two main challenges in the quality of water that we are dealing with worldwide: the lack of water sources in certain regions of the world and the consumption of contaminated water by at least two billion people, according to the World Health Organization (WHO) [1]. This means that these lives are endangered, contaminated water being tightly connected with the transmission of diseases such as cholera, diarrhea, type-A hepatitis, typhoid fever, dysentery, and polio. Diarrhea causes approximately 500,000 deaths each year [2,3]. In addition to this, contaminated water or water which has inadequate characteristics represents a main factor for the triggering or worsening of kidney diseases. Each year, kidney diseases cause as many deaths as those caused by cancer [3]. An important part of the population doesn’t consume bottled water, choosing public fountains, water pumps, or natural water sources in mountain areas. Alternative sources could be unsafe, because they are tested occasionally or never. Water quality and purity is a problem in different parts of the world, and different countermeasures and sustainable scenarios are being developed [4,5]. Different pollutants being present in water may also present a threat to the integrity of ecosystems. These affect plants and animals by compromising them, as well as by facilitating the development of viruses and bacteria. These in turn end up reaching human beings and are the causes of various diseases [6].

From another perspective, by 2050, over 85% of freshwater sources will be needed for agricultural purposes [7]; therefore, the quality of water should be monitored and assured to be proper for this purpose [8]. In the past, the overutilization of natural resources, pollutants, and the lack of legal framework implementation have led to water pollution in different areas of the world. One such region where different improvements have been implemented and different projects are running is that of Timis County, in Romania [9,10]. Here, specific European Union laws are being implemented, which is why water pollution has begun to be addressed by several approaches.

As the importance of monitoring and preserving the water quality in a large spectrum of sources has been largely highlighted, advanced but also low-cost technologies are used in the process. Inspired by these worldwide problems and statistics, as well as the support that retaining water quality receives from the European Union and local administrations, we have developed the idea of implementing a simple low-cost solution for water testing which will also keep people informed about the quality of water they are drinking. Correct information can prevent diseases, save lives, and improve quality of life. Our aim is to focus on rural areas in Romania, which is why our experiments have been conducted in the Gataia locality in Timis County. As the integration of water monitoring solutions has offered different architectural and technical solutions over the past several years, our aim is to validate existing knowledge from different scientific literature in terms of technical approaches, communication technologies, test conditions, lessons learned, and limitations in order to produce a low-cost approach that could be used in different rural areas which are currently on the verge of an increased developing slope. The obtained results offer a new perspective on the subject of water pollutant measurements, with respect to technical implementations, methodologies, and limitations, as well as highlighting the importance of such systems being developed in certain regions such as the one under study.

Our research aims to achieve several goals, highlighted here as the research questions of the paper: (RQ1) implement a water prototype internet-of-things system based on a low-cost board and different sensors; (RQ2) design the system to be controlled and managed from a mobile application, which will monitor the actual status of water sources, also offering the possibility of retesting them and offering updates, information, and recommendations to users in a real-time manner; (RQ3) monitor and evaluate the quality of water from different water sources, in a rural settlement, by taking into consideration specific measurement metrics. Our personal motivation for writing this present paper is to offer to the research public our experience of developing this system in a rural area that might benefit.

The rest of the paper continues: Section 1.1 presents basic concepts on the measured parameters used in this project; Section 2 presents state-of-the-art literature on water monitoring and specific techniques and systems developed for this goal, followed by Section 3 detailing the methodology used for our approach, as well as the proposed solution; Section 4 presents the experiments conducted to determine the quality of water sources in a certain locality, while Section 5 is reserved for the conclusions of our present research.

### 1.1. Measured Parameters

The pH of a solution is the negative of the logarithm of the hydrogen ion activity:

(1) pH=−log⁡(H+)

Measuring the pH of water provides a reflection of the acid–base balance, which is usually determined by the balance between carbon dioxide and bicarbonate carbonate [11]. The pH value increases if the concentration of carbon dioxide decreases and will decrease otherwise. Another factor that affects the pH balance is temperature; this must be taken into account when we take measurements. The pH of drinking water must have a value between 6.5 and 8.5.

A low pH of water causes the potential level of corrosion to increase; thus, the health effects are indirect through an increased ingestion of metals from plumbing or pipes and inadequate disinfection due to the lower pH. For water to be effectively disinfected with chlorine, the pH must be less than 8.

Water turbidity is a measure of the amount of light spread by the matter in a liquid when a light shines, making it an optical characteristic. The turbidity increases with the intensity of the light. Turbidity is caused by organic matter with very small dimensions, various colored organic compounds as solutes, plankton, and microscopic organisms.

The unit of measurement for turbidity is the nephelometric turbidity unit (NTU) and the optimal value for drinkable water is below 1 NTU, according to the WHO. However, if decontamination is proven, a turbidity with values less than 5 NTU is permitted. Water turbidity offers a favorable medium for the development of germs. This can lead to the occurrence of pest holes for illnesses that primarily affect the intestines. Research shows that there is a strong connection between removing the turbidity of the water and removing the protozoans.

Total dissolved solids (TDS) is the term used to describe the inorganic salts and small amounts of organic matter present in solution in water. The principal constituents are usually calcium, magnesium, sodium, and potassium cations and carbonate, hydrogen carbonate, chloride, sulfate, and nitrate anions [12]. Total dissolved solids are measured as water volume using the unit mg/l, also known as parts per million (ppm).

The TDS level has been classified according to the taste of drinkable water, as follows:

- Excellent: <300 ppm;
- Satisfactory: 300–600 ppm;
- Suitable/appropriate: 600–900 ppm;
- Scarce: 900–1200 ppm;
- Inadmissible: >1200 ppm.

According to the health recommendations for people, the classification is as follows:

- Between 50 and 150 ppm—excellent for consumption;
- 150–250 ppm—good;
- 250–300 ppm—equitable;
- 300–500 ppm—scarce;
- >1200 ppm—inadmissible.

The maximum level accepted for drinkable water is regulated by the EPA—Environmental Protection Agency—at a maximum of 500 ppm.

## 2. Previous Work

With both ground and surface water representing a vital resource for humans and other living species, a constant focus is kept on measuring the pollutants that might affect such a resource. The continuous development of IoT solutions during the last decade allows a real-time measurement of the water-quality levels for a large spectrum of applications. The following literature review was conducted based on research papers published after 2017, addressing the importance of water-quality measurement and providing various solutions. The succeeding paragraphs present the identified articles, grouped based on the described water-quality monitoring applications and solutions.

### 2.1. Specific Water-Quality Monitoring Applications

While assiduously analyzing the selected research papers, specific water-quality monitoring applications were identified which are reviewed below. In their work, Hafeez et al. [13] address the importance of monitoring case-II classified coastal waters. The authors focus on Hong Kong coastal waters and propose a solution for improving water-quality estimates by using machine learning. It has been concluded that the most accurate quality indicators were provided by the artificial neural network machine-learning model and satellite data. Similarly, Zompanti et al. [14] concentrated on monitoring the sea water iodide for assuring the quality of seafood. A multi-sensor approach was proposed to evaluate the seawater iodate level and thus to assess the quality of seafood. Their study proved the feasibility of the proposed proof-of-concept. Lu et al. [15] compared nine different machine-learning algorithms and using an unmanned aerial vehicle (UAV) borne for data collection. Concluding that the Catboost regression model offers the best prediction, a map was generated in order to identify polluted water areas. Likewise, Liu et al. [16] designed a UAV for air–water-quality monitoring activities of the Yangtze River. With a Chl-a distribution and a monitoring method based on acousto-optic tunable (AOTF) technology, the UAV-borne has been proven to be a reliable tool for water-quality spectral analysis. A multi-function unmanned surface vehicle (MF-USV) was designed and presented by Chang et al. [17] for water-quality analysis and water surface cleaning. Several other functions such as obstacle detection and avoidance were implemented and presented in the paper with experimental results verifying their effectiveness. In their paper, Tang et al. [18] presented a water intake monitoring system for livestock based on Arduino development boards. The proposed system can monitor the water quality but also track and identify each animal in order to conduct further animal water consumption studies. Another solution based on UAC and IoT for monitoring livestock was presented by Behjati et al. [19]. Microwave sensors were used by Frau et al. [20] to detect trace metals in polluted mining area waters. The authors described a novel technique making use of both microwave spectroscopy and planar sensors to monitor the water quality in real time. A similar approach was followed by Russul et al. [21] who used microwave sensors, similar to a microwave resonator, to detect the water content in crude oil. The obtained results of the experiments were found to be in line with the associated simulated results. An interesting approach was followed by Zeng et al. [22], who proposed a digital camera colorimetry setup that is able to examine the relationship between water color and its chemical composition.

### 2.2. Groundwater-Quality Monitoring

The following research papers propose various solutions for monitoring groundwater-quality levels. Shadrin et al. [23] present in their work a technique for computing the weighted water-quality index and to further provide a map containing area predictions of the water-quality index. Their proposed technique was validated in New Moscow where drinking water is provided mainly from the available groundwater. Another groundwater-quality mapping approach was described by Lawrence et al. [24] for a small island area of the Philippines, using a neural network with the particle-swarm optimization method. A machine-learning approach was used by Eslam A. et al. [25] in order to predict the availability of groundwater. The proposed solution including a global feature resulted from a Gaussian mixture model has proven to provide lower errors in groundwater-availability prediction. Focusing again on groundwater-quality assessment, Paepae et al. [26] reviewed the feasibility of using data-derived virtual sensors as an alternative to real-time sensor technologies. A review of the state-of-the-art literature was conducted underlining the necessity of developing a comprehensive virtual sensing system for IoT environments. Machine-learning methods were used for the first time by Mosavi et al. [27] to predict the hardness of groundwater. In their work, a comparative study was conducted between boosted regression tree and random forest machine-learning models.

### 2.3. Surface Water-Quality Monitoring

A case study conducted by Ferencz and Dawidek [28] focuses on the water quality of a Polish polymictic lake. Described as a critical lake management activity, monitoring vertical and horizontal variability values of the water must be conducted for assuring aquatic life. The water-monitoring method used—based on a nearest neighbor—proved to be more accurate in highlighting the fluctuation of the physical and chemical lake water parameters. Similarly, a case study was conducted by Ouali et al. [29] focusing on the Hassan Addakhil dam in Morocco. Using a modeling approach and remote sensors, the authors propose a spatiotemporal water-quality measuring technique. The selected and presented approach has provided results validating the methodology for mapping the quality of the water reservoir. By using a Raspberry Pi, Budiarti et al. [30] proposed an intelligent and automated surface monitoring system that is capable of transmitting real-time information. In their published work, Zhao et al. [31] aimed to understand whether third-party water-quality monitoring activities have improved China’s environmental data. Even with a certain amount of discontinued data monitoring, the research proved that third-party organizations have helped in the reduction in data manipulation of local governances and ensured more accurate and consistent water-quality environment data. Zhu et al. [32] proposed a solution for monitoring the dynamic water changes in Poyang Lake. The authors developed an algorithm based on texture feature, feature fusion, and target segmentation for a synthetic aperture radar system that was used for water-quality monitoring. The results were proven to be accurate as they were compared to actual hydrological data. Naloufi et al. [33] evaluated the various models that have been developed to predict the microbial quality of surface waters and provide a guideline for choosing the appropriate machine-learning model and sampling. Rodriguez et al. [34] assessed several machine-learning models for water-quality detection at Santa Lucia Chico river stations. The proposed approach of their research paper is now expected to help in improving water-quality datasets and therefore overcome missing important datasets. The importance of bettering the image special resolution and mitigating the interference of mixed pixels in cases of monitoring the water quality of small rivers are highlighted by Huangfu et al. [35]. The processed Setinel-2 images using the super-resolution algorithm have proven the potential of the algorithm in improving the retrieval accuracy of several water-quality parameters.

### 2.4. Potable Water-Quality Monitoring

Many resource papers provide different solutions for monitoring potable water, as it is a vital source of life. Contaminated water leads to millions of deaths each year and it is the cause for many diseases, so the following solutions are life-saving.

Wong Jun Hong et al. [36] published a simple Arduino-based solution with multiple attached sensors that has some inaccuracies and needs human assistance, but it is a foundation for future works. Sami O. Osman et al. [37] also used Arduino and sensors for designing an in situ real-time measurement system for water-quality parameters, which is a low-cost and accurate solution. Going further, a similar system that uses Bluetooth to send the values of water parameters on a smartphone was built by Chenwei Feng et al. [38].

Likewise, Yogesh K. Taru and Anil Karwankar [39] implemented a solution for water-parameter monitoring interfacing Arduino with LabVIEW. As a more advanced technological approach, Ms. Ch. Sowmya et al. [40] described in their paper a solution with wireless sensor network technology for online and real-time water-quality monitoring, where each sensor node has an Arduino microcontroller and attached sensors that continuously measure the most important water parameters—pH, temperature, and conductivity. Irish Franz Almojela et al. [41] also used WSN in their published work, using two nodes and displaying the data on an LCD and ThingSpeak channel. The water parameters and alarm are sent as an SMS notification and, for an alarm, the system also integrated a buzzer. Another real-time system was proposed by Budiarti et al. [30] using a Raspberry Pi, as mentioned earlier.

Various real-time systems are implemented using the concept of IoT, such as the one proposed by Sabari et al. [42] that used an Arduino as a microcontroller and a Wi-fi module for viewing the water-parameter values on the cloud. In addition, Ali Hadi Abdulwahid [43] proposed a similar solution, using the same IoT concept and accomplishing the visualization of data on the cloud. Dr. Nageswara Rao Moparthi et al. [44] have published a solution for notifying the corresponding authorities about water contamination using Arduino. For the message technique, they used a GSM module and extended the work by sending the sensor data via the cloud. L. Lakshmananet al. [45] also used a GSM module connected to Arduino for measuring water-quality parameters with sensors and displaying the results on a webpage. Data were also stored on the cloud so others could access it.

Rajesh Singh et al. [8] went to the next level, using an industrial IoT with hardware architecture designed for water-level measuring and quality that uses radiofrequency communication and a cloud-enabled app for visualizing the data. Another approach for monitoring potable water was described in Arif Ul Alam, Dennis Clyne, and M. Jamal Deen’s resource paper [46]. They developed a low-cost multi-parameter water-quality monitoring system, which uses high-sensitivity electrochemical sensors and custom-designed circuitry that communicates with an Android app. This is a much more affordable testing system than testing the water in dedicated laboratories and can be easily programmed and adapted.

### 2.5. Machine Learning for Water-Quality Monitoring

Machine learning has been a huge step in technology, as it provides futuristic and efficient ways in detecting, monitoring, and improving water-quality systems. In our research, we have found various solutions and important work that has been published in that direction.

An interesting solution for calculating a water index based on inputs such as temperature, pH, total dissolved solids, and turbidity using different machine-learning algorithms was published by Ahmed Umair et al. [47]. Their conclusion shows that the best precision for the water index is achieved using these two algorithms: gradient boosting, with a learning rate of 0.1; and polynomial regression, with a degree of 2. Likewise, we have mentioned in a previous section the study by Lu et al. [15] that also tested machine-learning algorithms for water testing.

Machine-learning techniques were also used by Hussein Eslam et al. [25] and Huangfu Kuan [35] in their studies on groundwater, as previously mentioned. For predicting handover events based on historically collected handover events, Esraa Eldesouky et al. [48] used machine-learning algorithms in an underwater wireless sensor network.

Another very interesting study can be found in the work of Arias-Rodriguez et al. [49], who conducted a case study in Mexico that uses remote-sensing-based machine-learning approaches for estimating the water-quality parameters. They used in situ collected measurements and remote-sensing reflectance data gathered from MERIS—the medium resolution imaging spectrometer, demonstrating the utility of satellite observations in monitoring inland water. Similarly, also in Mexico, Leonardo F. Arias-Rodriguez et al. [50] in their paper conclude that remote sensing is very useful for monitoring and can bring improvements in water-quality monitoring programs due to its progressive integration. Remote-sensing using machine-learning techniques was also used in the case study of Hafeez Sidrah et al. [13] in Hong Kong for estimating coastal water quality. A higher accuracy was achieved by an artificial neural network.

On the other hand, Pu Fangling et al. [51] proposed an alternative for solving the issues that remote sensing may have, using a convolutional neural network (CNN) with a hierarchical structure that represents the relationship between Landsat8 images and in situ measurements. They successfully demonstrated that CNN widely outperforms the machine-learning methods. Other machine-learning models have been evaluated by Naloufi et al. [33] for microbial water quality and by Rodriguez et al. [34] for water-quality detection: we have previously described their approaches.

Stajkowski Stephen et al. [52] suggested a premier approach in this domain, as they sustain in their paper, that uses a genetic-algorithm-optimized long short-term memory (LSTM) technique to predict river water temperature. Their goal was to use advanced machine-learning methods in order to create a tool that is compatible with a real-time network of water-quality monitoring stations for proactive water-quality management.

Lastly, a very interesting resource paper was published by Post Claudia et al. [53] which can be used in order to detect chemical pollutants, such as nitrates, pharmaceuticals, or microplastics. The study uses a UV Raman spectrometer for the detection of nitrate/nitrite, selected pharmaceuticals, and the most widespread microplastic polymers. The results show that nitrates and nitrites can be detected and quantified but the microplastic particles measurements suffer due to their heterogeneous distribution.

A detailed comparison of different studies from all the references we have studied is presented in Table 1. This summary is also offering insights into comparing previous studies with the current approach.

## 3. Materials and Components

**Figure 1:**
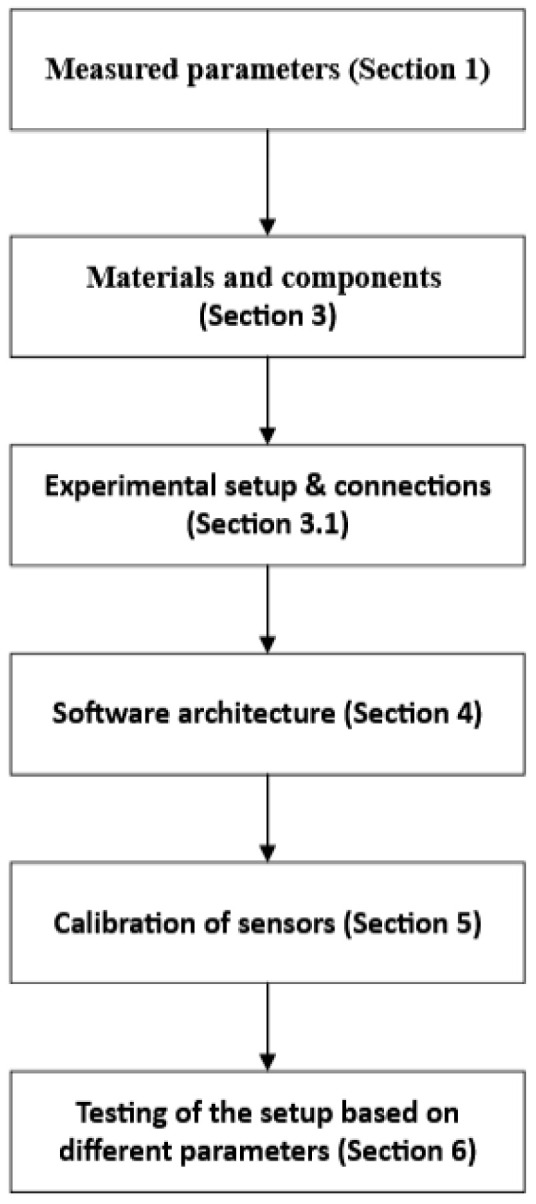
Research methodology.

The system presented in this paper offers a prototype implementation of an internet-of-things water-testing solution based on an Arduino UNO board and different sensors. The research methodology (Figure 1) that our project implemented comprises the following steps:

1. Discuss the measured parameters that the project is aiming at (Section 1);
2. Build a low-cost prototype capable of measuring different water parameters from different water sources (Section 3 and Section 3.1);
3. Implement a mobile application to receive data from the sensors, such as the DS18B20 temperature sensor, SEN0161 pH sensor, TDS SEN0244 sensor, and turbidity sensor SKU SEN0189 (Section 4);
4. Calibrate the sensors in order to obtain accurate values from different measurements (Section 5);
5. Perform the measurement of different water parameters from several water sources in a rural setting (Section 6);
6. Analyze and discuss the obtained results, as well as make the results available to the public through their apps (Section 6).

The system will be controlled and managed from a mobile application, which will monitor the actual status of water sources, also offering the possibility of retesting them and offering updates, information, and recommendations to users in a real-time manner. We have performed a case study for the Gataia locality, in the western part of Romania, by testing five water sources, which represent the main drinking and cooking sources for locals. The water status system and application have the purpose of informing people and protecting people through information, prevention messages, recommendations, and alerts regarding water consumption from drinkable water sources. The retesting of water sources at short periods of time is also facilitated through the application’s interface. The system only requires the power-up of the Arduino board and the submergence of the sensors into the water. The application is responsible for the other tasks such as receiving the values via Bluetooth, database updates, and users’ notification.

### 3.1. Setup and Connections

**Figure 2:**
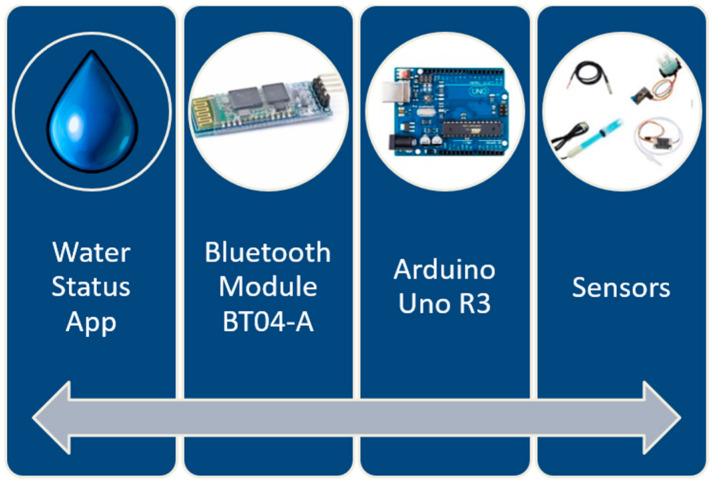
System architecture of the water status project.

In order to understand the functioning of the entire system, including the mobile application, we present the general architecture of the system below (Figure 2). The water status application communicates with the system composed of an Arduino board, sensors, and a Bluetooth module. The Bluetooth module ensures communication between the hardware system and the Android mobile application.

**Figure 3:**
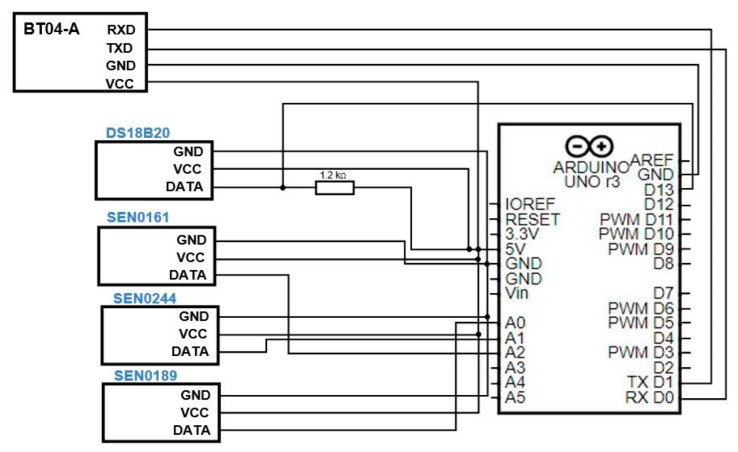
The hardware architecture of the water status project.

**Figure 4:**
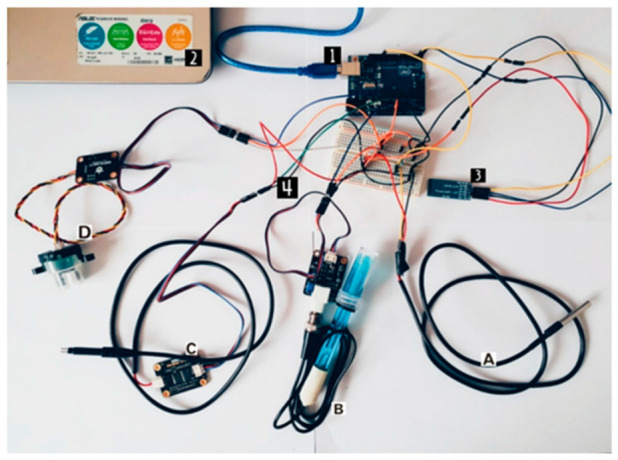
Setup of the Arduino-based sensor system.

The hardware architecture (Figure 3) is composed of several peripherals. These can be noted in Figure 4, which presents the physical connections of the hardware components. Our approach is orientated towards a low-cost system architecture which could be afforded by anyone who would like to perform measurements. The prices for the components are presented in Table 2 and the list of peripherals is:

1. Arduino microcontroller (label 1, in Figure 4);
2. Power source (label 2);
3. BT04-A Bluetooth module (label 3) for data transmission to the dedicated mobile app;
4. Sensors—DS18B20 temperature sensor (label A), SEN0161 pH sensor (label B), SEN0244 TDS sensor (label C), and turbidity sensor SKU SEN0189 (label D).

The connections of the system are as follows:

- Arduino board: connected through a USB cable to a power source (or laptop/PC);
- Bluetooth BT04-A module: GND to Arduino GND (black wire), VCC to 5 V (red wire), Tx to Arduino Rx—pin 0 (blue wire), Rx to Arduino Tx—pin 1 (yellow wire);
- DS18B20 temperature sensor: connected to GND (black wire), to 5 V (red wire), and to pin 13 of the Arduino board (yellow wire), by using a 1.2 KΩ resistor;
- SEN0161 pH sensor: connected to GND (black wire), to 5 V (orange wire), and to pin A2 of the Arduino board (blue wire);
- TDS SEN0244 sensor: connected to GND (black wire), to 5 V (red wire), and to pin A1 of the Arduino board (green wire);
- SKU SEN0189 turbidity sensor: connected to GND (black wire), to 5 V (orange wire), and to pin A0 of the Arduino board (blue wire).

As can be noted in Figure 3 and Figure 4, we have used four sensors in order to collect relevant data about water quality:

- The DS18B20 temperature sensor is a programmable sensor with a single wire and it is used for measuring the temperature in different environments; due to its waterproof case, it is used for measuring the water temperature. This sensor is easy to use and its connection to the Arduino board requires only one data pin, along with a pull-up resistor.
- The SEN0161 pH sensor is an analog sensor for pH measurement, which is particularly designed for Arduino development boards, featuring simple characteristics and connections. It contains an LED for supply voltage indication, a BNC connector, and a PH0.2 interface.
- The TDS SEN0244 sensor (for total dissolved solids) is also an analog sensor, easy to use, and Arduino-compatible, which measures the TDS value of water and it transmits it to the system.
- The turbidity sensor SKU SEN0189 is also an analog sensor, easy to use, and Arduino-compatible. This offers a response depending on the light amount that passes through a liquid, thereby being able to detect the number of total suspended solids—TSS.

## 4. Software Architecture

**Figure 5:**
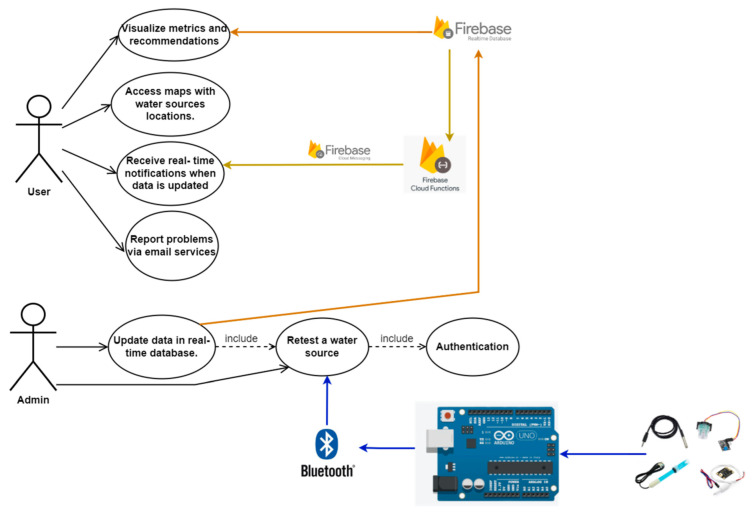
Software architecture of the water status mobile application.

In order to describe the software architecture of the application, we will define each module separately. An overall schematic of the architecture is presented in Figure 5, which contains the use cases of the application, as well as the role of the integrated technologies and the role of the hardware system.

### 4.1. Water Status Mobile Application

By using the water status application, the users can check the status of all potable water sources from the city. The parameters that the application will provide will be the pH level, the total dissolved solids, and the water turbidity. In addition, recommendations or alerts will be issued according to these metrics. In the case of extreme measured values, a warning message will be displayed, in order to prevent the consumption of water from that source. The users will be provided with a map where the drinkable water sources are displayed. When the water source is being retested, the user will be notified of this in real time. The notification becomes very useful when a water source becomes contaminated due to acid elements from the rain or ecological accidents that affect the underground water.

The administrator of the application has a key role because they should periodically retest the water sources, in order to provide relevant and up-to-date data. The administrator will have an account in order to log in. This secure access will prevent unauthorized access. For the locality of Gataia, this role could be fulfilled by the hydrotechnical engineer, who is responsible for the region’s rivers and the drinkable water sources.

Through Bluetooth communication, when pressing a button in the application, the Arduino-based system reads the values from the sensors about the tested water metrics and displays them for the administrator. The administrator can also restart the sensors’ readings in order to ensure that the values are real and error-free. The application also offers the feature of updating the database. All users will be notified about the retesting of the water source.

**Figure 6:**
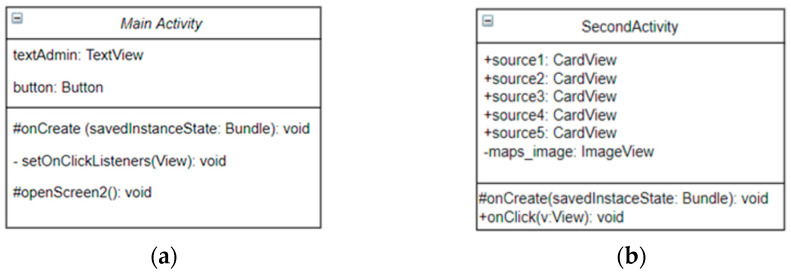
(**a**) Class diagram of main activity of the mobile application; (**b**) class diagram of the second activity of the water status app.

The main activity of the application (Figure 6a) allows the user to reach the secondary activity, which will offer the possibility to choose between the drinkable water sources of interest. A common user doesn’t need an account to log in in order to access the application because this application should be used by people belonging to all age categories. Similarly, starting from the main activity, the admin will touch the text element of the TextView type, called textAdmin, which will redirect them to another authentication activity. For the administrator, this security feature is required through authentication because they are responsible for the retesting of the water sources and updating the database, where the access of other users is not permitted.

**Figure 7:**
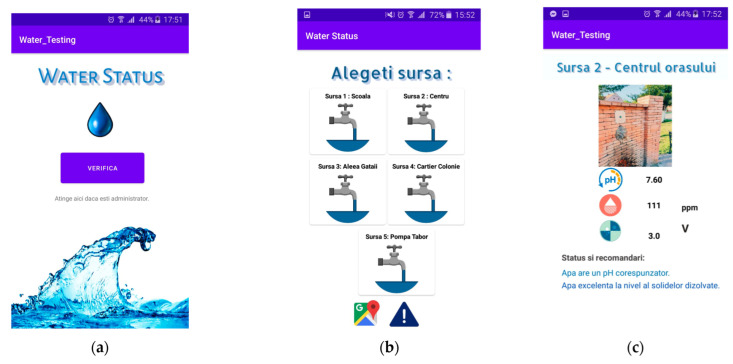

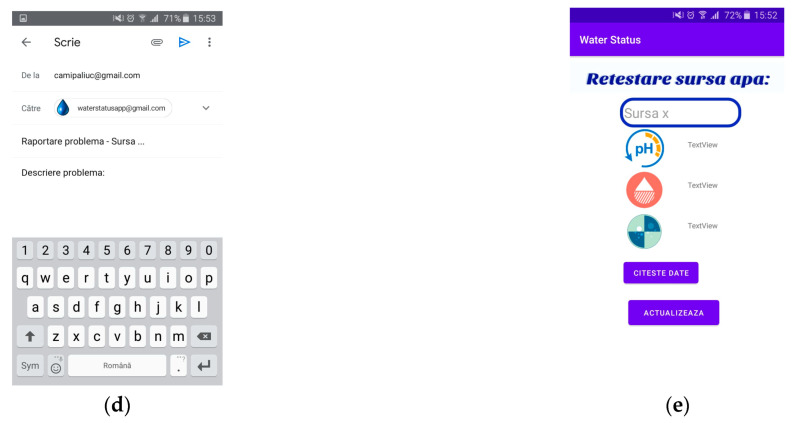
Graphical user interfaces for: (**a**) the main activity; (**b**) a certain water source; (**c**) finding the water sources in the locality; (**d**) issue reporting; (**e**) water-source retest activity.

The second activity (Figure 6b) which can be viewed by the user contains CardView-type elements associated with each drinkable water source from Gataia town. The user can select whichever card among the five, in order to view detailed information about that water source (Figure 7). When selecting one of the cards, a new activity is initiated associated with that water source. For each of the five drinkable water sources of Gataia town, an individual activity was created, with associated classes: source one, source two, source three, source four, and source five. For example, the source one activity offers the user information about water source one, which is the water pump situated in front of the town’s school (101 Republicii street, Gataia, Timis county). The user can view the values for pH, TDS, and water turbidity. In addition, the status and consumption recommendations will be shown: the appurtenance to optimal standards values, the recommendations related to some illnesses, or general consumption recommendations.

Starting from the previously presented activity, by clicking the map icon, the user will be redirected to the present activity, where the water sources of Gataia locality will be marked on a map portion from Google Maps. A pin is added onto the map for each water source. When touching the marker, the number and name of the water source will be displayed and, by using Google Maps services, the user will be able to obtain navigation guidelines to one of the five sources.

Another functionality dedicated to the user is that for reporting an issue. Using an email service installed on a smartphone (Gmail, Yahoo), a template will be available in order to describe the issue, for example: the water from a certain source has a strange taste, an unusual color, or if some action that could contaminate the water has been observed.

For the administrator role, the authentication activity validates the username and the password. The credentials are unique and are integrated in the application (we assume that they are familiar to the hydrotechnical engineer responsible for the locality). After the authentication process takes place, the administrator will be redirected to the water source retesting activity.

**Figure 8:**
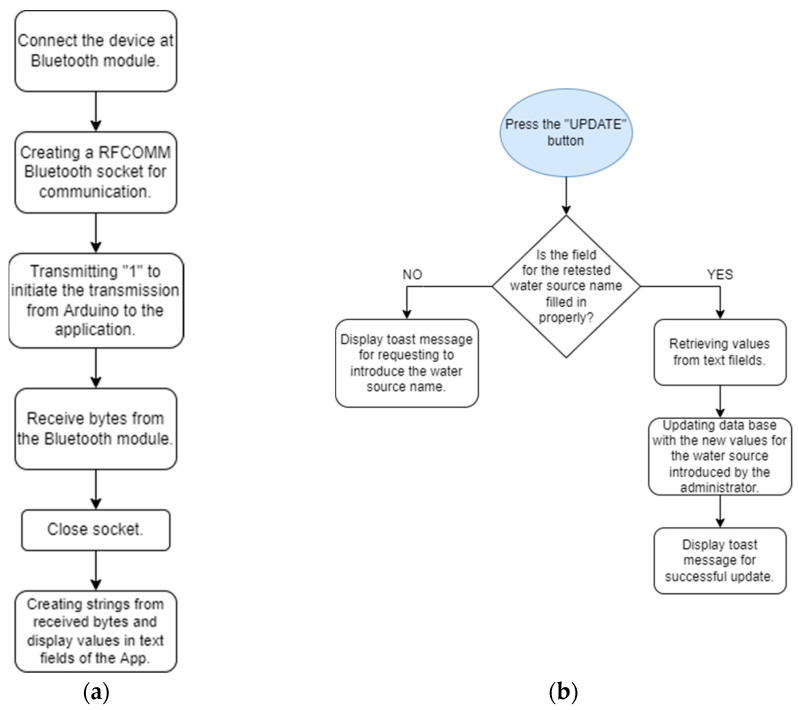
Operations performed in the: (**a**) readData() function and (**b**) updateData() function.

The retesting activity is conducted by reading the values from the sensors and updating the database in a real-time approach. Therefore, this activity performs two different functionalities which also depend on each other. These are implemented in two different functions, readData() and updateData() (Figure 8a,b).

In the readData() function, the connection to Bluetooth is established. In order to achieve this, an object of the type BluetoothAdapter is used. The process of managing the connectivity is implemented by using a BluetoothSocket object, using the most common RFCOMM (radio frequency communication) socket, which is a type supported by the Android APIs.

The values for updateData() will not be undertaken unless the text field—which suggests the entry of the number of the tested water source—is previously filled in. This is necessary in order to update the data of the correct reference in the real-time database (which is Firebase for the water status project). Upon obtaining the correct reference to indicate the water source, we will update the values using the function setValue (newvalue) for each parameter which is a child of the water source in the JSON tree structure of the database.

### 4.2. Software for the Arduino Board

**Figure 9:**
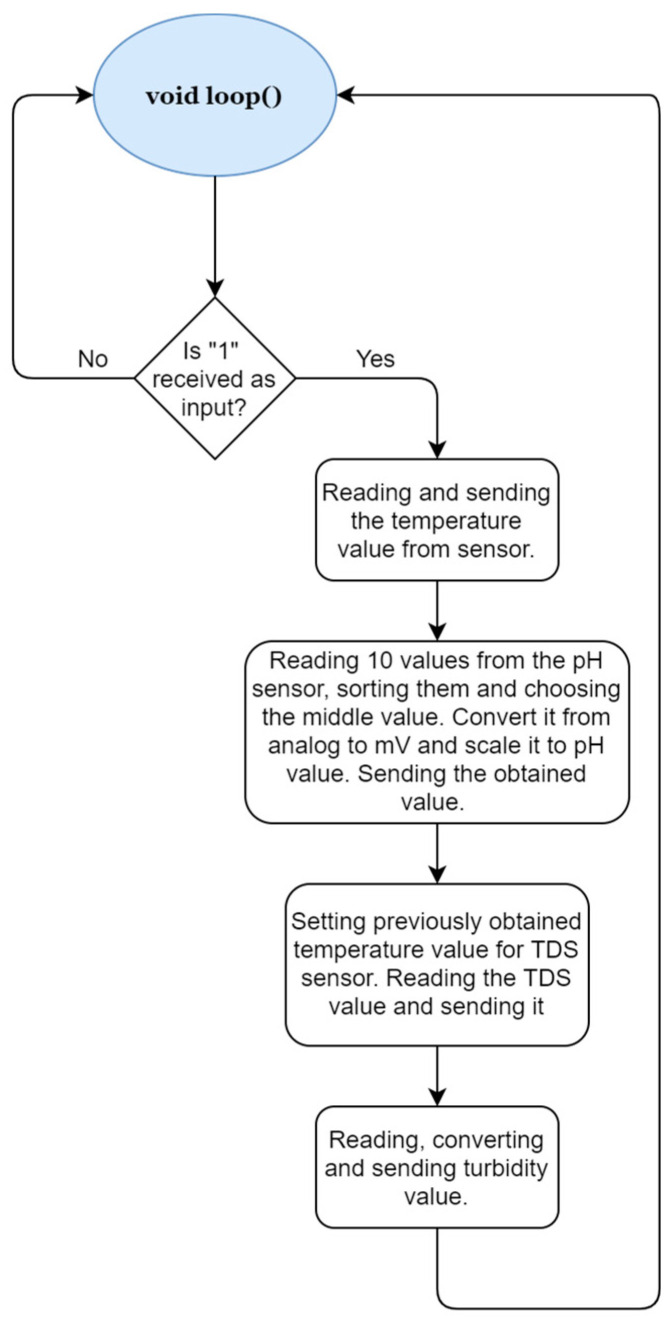
Diagram for the software implemented on the Arduino board.

The Arduino board collects the data from the sensors submerged in the water and transmits the data using the Bluetooth module towards the mobile application, when this is requested. The values from the sensors will be read only when this is requested by the administrator from the mobile app. The program will wait until it receives the value “1” via Bluetooth from the mobile app. This is transmitted from the mobile app to the system when the administrator presses the read data button, as can be noted in Figure 9.

After the request to transmit data from the sensors, the values from each sensor are read and transmitted: temperature sensors, pH, TDS, and turbidity. The temperature sensor is not relevant for the quality of the water but its value is required in the computation of the total dissolved solids.

## 5. Calibration of Sensors

In this subsection of the paper, we present the calibration process applied to our system. This is necessary in order to obtain error-free data from the physical measurements, which will be sent towards the Firebase database for visualization in the mobile application.

Calibration of the pH sensor implies two processes: first of all, the process of calibrating the sensor takes place, and secondly, the accuracy of the pH sensor is compared with a digital pH meter.

The steps to calibrate the pH sensor are as follows:

1. Prepare two buffer solutions with different pH. In the scientific literature [8], the minimum of this pH is 4.00, while the maximum we found to be either pH 7.00 or pH 9.20;
2. Dip the SEN0161 pH sensor in the pH 4.00 buffer solution;
3. Set the sensor to read the pH as having a value of 4.00;
4. Clear the probe with distilled water;
5. Dip the SEN0161 pH sensor in the pH 9.20 buffer solution;
6. Check the sensor reading;
7. Record the difference in the sensor reading, this is the error;
8. Clear the probe with distilled water;
9. Dip the SEN0161 pH sensor in the solution for which the pH should be obtained;
10. Calculate the actual pH with the following equation:
  (2) Actual pH=pH reading+Error
11. For the pH sensor value to be relevant and in accordance to reality, we have collected 10 successive values, we have sorted the vector, and we have used the average value. Hereafter, we have transformed this value in millivolts by using Equation (3).
  (3) $$Value=\frac{average\;value*5}{\frac{1024}{10}}$$
12. We have multiplied by 3.5 the value from step 11, in order to obtain the pH value.

By applying the previous steps to our setup, the resulting error was 0.30 for pH 4.00 and 0.60 for pH 9.20. We present the obtained data only for 0.30, and the final values will be highlighted in Table 3, Table 4 and Table 5 in Section 5.

The calibration of the TDS sensor follows a process similar to the one described for the pH sensor. In order to increase the accuracy in measuring the TDS values, a temperature sensor such as the DS18B20 could be used for temperature compensation in order to improve accuracy [54]. A liquid solution with a known electrical conductivity or a known TDS value is required. A standard buffer solution for this purpose will have about 707 ppm. Otherwise, the reference value could be measured by means of a TDS pen.

The steps to calibrate the TDS sensor are as follows:

1. Prepare a standard buffer solution as previously described or a TDS pen;
2. Dip the TDS SEN0244 sensor in the buffer solution;
3. Clear the probe with distilled water;
4. Dip the TDS SEN0244 sensor in the solution to be measured;
5. Check the sensor reading;
6. Record the difference in the sensor reading, this is the error;
7. Clear the probe with distilled water;
8. Calculate the actual TDS value with the following equation:

(4) Actual TDS=TDS reading+Error

We have applied the described steps to our setup and the resulting error was 13. The final value are presented in Table 3, Table 4 and Table 5 in Section 5.

## 6. Testing of the Setup

The water status system concentrates on testing the three parameters previously described. The tests were performed as a basis for developing the app on the five drinkable water sources of Gataia locality, Timis County in Romania, from which the majority of the population consumes drinkable water. The testing of these water sources was performed during multiple months, testing the same quantity of water from each source. Each water sample from the five sources was tested for 3 days in a row, because people replenish their water stocks every 3 days on average. Therefore, we wanted to observe whether the drinkable water from these sources changes its characteristics when stored. The collected data from the five sources are presented in Table 2, Table 3 and Table 4.

**Figure 10:**
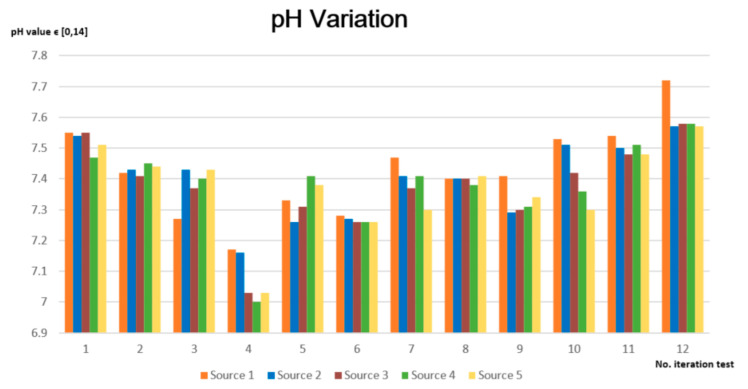
pH variation for the tested water sources.

The pH variations from Figure 10 demonstrate that each of the five sources have pH values situated between 6.5 and 8.5, considered to be the optimal interval. Therefore, the water from all sources is appropriate for consumption; we did not distinguish an acidic tendency of the water sample, nor an alkaline tendency.

For the total dissolved solids, the tests’ results demonstrate that one of the water sources outperforms not only the ideal values but also the maximum accepted value of 500 ppm, and this water source is source five—the Tabor water pump. Therefore, this water is not recommended for consumption because it can lead to intestine illnesses.

**Figure 11:**
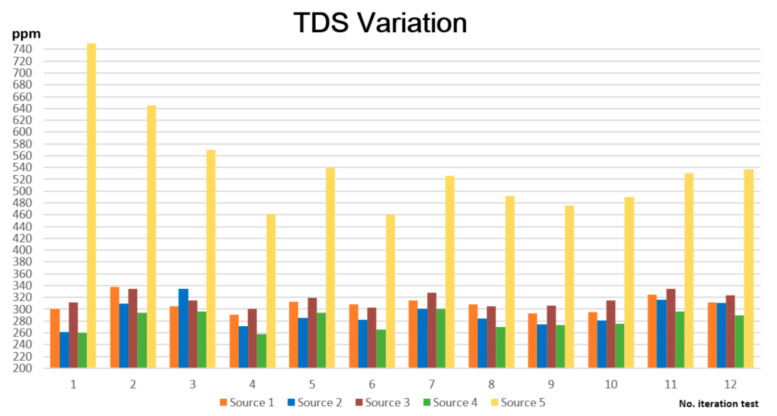
TDS variation for the tested water sources.

Regarding TDS (Figure 11), the best drinkable water is the one marked in purple, source four—the Colonie neighborhood, which has the lowest values—between 258 and 301.

Another conclusion that can be drawn is that none of the water sources belong to the category of excellent drinkable water, with a TDS value between 50 and 150 ppm. However, four of the five water sources have acceptable values, situated between 258 and 335 ppm.

**Figure 12:**
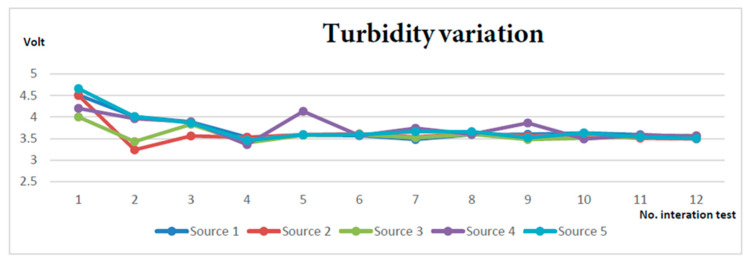
Turbidity variation for the tested water sources.

From the collected data, it can be observed (Figure 12) that the turbidity of all sources is quite linear with values close to each other.

The application and the measurement results have been publicized to the population of the rural settlement by means of direct discussions and leaflets. Specifically, the results from source five (Tabor) had the most important impact, as the TDS concentration proves that the water from this source is not recommended for consumption. A further campaign is planned to take place by contacting the local administration and presenting the application. Our approach is that the application will be advertised by the local administration in the local newspaper which is read by all the people from this rural area.

## 7. Conclusions

Water quality has been proven as a key factor for maintaining the populace’s health; therefore, different systems have been proposed in order to monitor and assure the quality of water. This paper addresses the necessity of monitoring water in the rural areas of Romania. Therefore, we propose a low-cost internet-of-things proto-type which can be used to detect different parameters from drinkable water. Our results demonstrate that our system can be expanded to other rural areas that might need water monitoring. It also shows which of the water sources from our experiments can be used for public consumption and which should be avoided. In our experiments, all the water sources are drinkable, with a single exception where the TDS values are not within proper limits, as they outperform the maximum accepted value of 500 ppm.

In future work, we plan to involve the local council of the rural area in promoting the benefits of the system, as well as the results. In the case of the water sources which are not drinkable, this should be announced to the population as fast as possible via different channels.

From the technical point of view, we plan to extend the number of sensors with another spectrum—specifically one which can measure different chemical compounds—as well as a method of data analysis based on several machine-learning approaches.

## Figures and Tables

**Table 1:** Findings of previous studies compared to the current approach.

Reference	Description	Addressed Points	Current Approach
[24]	The implementation of various spatial interpolation methods results in significant variations from the true spatial distribution of water quality in a specific location. This research improves the mapping prediction capabilities of spatial interpolation algorithms	Groundwater-quality mapping using a neural network approach	Water-quality monitoring system based on specific measured parameters
[26]	The feasibility of virtual sensing for water-quality assessment is reviewed. The review focuses on the overview of key water-quality parameters for a particular use case and the development of the corresponding cost estimates for their monitoring	Multiple solutions are analyzed in terms of modeling approaches and various parameters	The water-quality monitoring system is based on low-cost components
[30]	Web scrapping and Python scripts are used for analysis, aggregation, and filtering of data from the sensors	An integrated system based on internet-of-things for measuring the water quality; MQTT protocol communication; MariaDB databases; sensors’ data graph plotting	Firebase databases used for the development of the water status app
[34]	Several statistical and machine-learning models are used for imputing water-quality data at six monitoring stations	Water-quality data analysis using a machine learning approach	Water-quality monitoring system based on specific measured parameters. The machine learning approach is part of future work
[35]	This study tries to improve the image spatial resolution and to weaken the interference of mixed pixels in the image. The study is focused on the water-quality monitoring of medium- and small-sized inland rivers	Remote estimation of water-quality parameters using Sentinel-2 imagery	The measured parameters: pH, turbidity, TDS
[41]	Arduino-based monitoring system that measures four physicochemical parameters of water: pH, temperature, turbidity, and electrical conductivity in order to identify possible water contamination	WatAr: an Arduino-based drinking water-quality monitoring system using a wireless sensor network and GSM module	The communication used in our approach is based on Bluetooth module BT04
[43]	Affordable system to control water quality in real time, based on several sensors, which measure various chemical and physical water properties, such as conductivity, pH, turbidity, and temperature.	Real-time water-quality monitoring using various sensors	The measured parameters: pH, turbidity, TDS
[8]	An IoT-based architecture is proposed and implemented for monitoring the level and quality of water in a domestic water tank with customized hardware based on 2.4 GHz radiofrequency (RF) communication	Real-time experimental setup for water-quality monitoring	The communication used in our approach is based on Bluetooth module BT04 and a Firebase database
[46]	A multi-parameter water-quality monitoring system (MWQMS) is proposed that includes an array of low-cost, easy-to-use, high-sensitivity electrochemical sensors, as well as custom-designed sensor readout circuitry and a smartphone application with wireless connectivity	Simultaneous monitoring of pH, free chlorine, and temperature with high levels of sensitivity	The measured parameters: pH, turbidity, TDS

**Table 2:** Components prices for a low-cost system approach.

Components	Price (in Euro)
Arduino UNO r3	39
Bluetooth module BT04	1.4
Temperature sensor DS18B20	2.4
pH Sensor—SEN0161	44.4
TDS Sensor—SEN0244	17
Turbidity Sensor—SKU SEN0189	16
Total	120.2

**Table 3:** Water source one and two results.

Date	Source 1—School				Source 2—Center			
	Temperature (Grade)	pH	Turbidity (V)	TDS (PPM)	Temperature (Grade)	pH	Turbidity (V)	TDS (PPM)
27 October 2022	21.37	7.55	4.51	300	20.87	7.54	4.50	261
29 October 2022	22.00	7.42	4.01	338	22.06	7.43	3.24	309
1 November 2022	22.94	7.27	3.89	305	23.00	7.43	3.56	335
12 November 2022	28.00	7.17	3.53	291	27.37	7.16	3.53	271
13 November 2022	22.69	7.33	3.58	313	22.50	7.26	3.59	285
14 November 2022	21.19	7.28	3.57	308	21.31	7.27	3.61	282
26 November 2022	23.56	7.47	3.48	315	23.87	7.41	3.54	301
27 November 2022	22.94	7.40	3.59	308	22.81	7.40	3.62	284
28 November 2022	24.56	7.41	3.60	293	24.81	7.29	3.56	274
4 December 2022	23.37	7.53	3.63	295	23.37	7.51	3.58	281
5 December 2022	23.12	7.54	3.59	325	23.19	7.50	3.51	316
6 December 2022	23.00	7.72	3.54	312	22.94	7.57	3.49	310
Average:	23.22	7.42	3.72	305.5	23.17	7.39	3.61	292.4
Minim:	21.19	7.17	3.48	291	20.87	7.16	3.24	261
Maxim:	28.00	7.72	4.51	338	27.37	7.57	4.50	335

**Table 4:** Water sources three and four results.

Date	Source 3—Gataia Street				Source 4—Colonie Neighborhood			
	Temperature (Grade)	pH	Turbidity (V)	TDS (PPM)	Temperature (Grade)	pH	Turbidity (V)	TDS (PPM)
27 October 2022	20.75	7.55	2.41	300	20.87	7.54	4.50	260
29 October 2022	21.94	7.41	3.43	338	22.06	7.43	3.24	294
1 November 2022	23.00	7.37	3.83	305	23.00	7.43	3.56	296
12 November 2022	27.97	7.03	3.40	291	27.37	7.16	3.53	258
13 November 2022	22.62	7.31	3.58	313	22.50	7.26	3.59	294
14 November 2022	21.19	7.26	3.59	308	21.31	7.27	3.61	266
26 November 2022	23.81	7.37	3.52	315	23.87	7.41	3.54	301
27 November 2022	22.87	7.40	3.60	308	22.81	7.40	3.62	270
28 November 2022	24.94	7.30	3.48	293	24.81	7.29	3.56	273
4 December 2022	23.00	7.42	3.51	295	23.37	7.51	3.58	275
5 December 2022	23.12	7.48	3.54	325	23.19	7.50	3.51	296
6 December 2022	22.94	7.58	3.57	312	22.94	7.57	3.49	290
Average:	23.17	7.37	3.45	308.5	23.17	7.39	3.61	281
Minim:	20.75	7.03	2.41	291	20.87	7.16	3.24	258
Maxim:	27.97	7.58	3.83	338	27.37	7.57	4.50	301

**Table 5:** Water source five results.

Date	Source 5—Tabor			
	Temperature (Grade)	pH	Turbidity (V)	TDS (PPM)
27 October 2022	21.87	7.51	4.66	753
29 October 2022	22.19	7.44	4.01	645
1 November 2022	23.31	7.43	3.86	570
12 November 2022	27.87	7.03	3.46	461
13 November 2022	22.56	7.38	3.59	539
14 November 2022	21.31	7.26	3.59	460
26 November 2022	23.94	7.30	3.66	526
27 November 2022	23.12	7.41	3.66	492
28 November 2022	24.81	7.34	3.52	476
4 December 2022	23.25	7.30	3.63	490
5 December 2022	23.44	7.48	3.54	530
6 December 2022	23.06	7.57	3.50	537
Average:	23.39	7.37	3.72	539.9
Minim:	21.31	7.03	3.46	460
Maxim:	27.87	7.57	4.66	753

## Data Availability

Data sharing not applicable.
